# Development of a Comprehensive Chinese Successful Aging Scale: Incorporating the Viewpoints of Older Adults

**DOI:** 10.1093/geroni/igab046.827

**Published:** 2021-12-17

**Authors:** Edwin K H Chung, Dannii Yeung

**Affiliations:** City University of Hong Kong, Hong Kong, Hong Kong

## Abstract

Inspiring by Martinson and Berridge’s (2015) systematic review, the current definition of successful aging (SA) fails to acknowledge the laypeople’s conceptualization of SA. Adopting a mixed-method approach, two studies were conducted with the aim of soliciting older adults’ perceptions of SA and to develop a multidimensional instrument for assessing SA. Study 1 was a qualitative study and 27 community-dwelling older adults (Mage=68.07 years, SD=7.10, range=60–83; 56.3% females) were interviewed. Interview transcripts were analyzed, and seven themes were emerged. An initial item pool for the Successful Aging Scale (SAS) was then established based on these themes as well as those in the SA literature, such as acceptance and independence. Study 2 was a survey study which was conducted among 414 community-dwelling older adults (Mage=64.50 years, SD=4.01, range=60–82; 55.3% females) to identify optimal items for constitution of the SAS. Exploratory factor analysis revealed a 12-factor solution, accounting for 62% of the variance. The 12 factors are adequate health, perceived constraints, flexible attitudes toward life, acceptance of age-related change, life embracement, active engagement, harmonious family, supportive friendship, civic awareness, social contribution, living independently, and adaptive coping strategies. The 12 factors exhibit similar strength of associations with most of the well-being measures, but certain factors show stronger correlation with depressive symptoms and social relationship, suggesting the uniqueness of each factor. Overall, the SAS demonstrates promising psychometric properties. These findings disclose that the older adults’ perceptions of SA could cover broader dimensions than those in Rowe and Kahn’s model (1997).

